# BNIP3 Regulates AT101 [(-)-Gossypol] Induced Death in Malignant Peripheral Nerve Sheath Tumor Cells

**DOI:** 10.1371/journal.pone.0096733

**Published:** 2014-05-13

**Authors:** Niroop Kaza, Latika Kohli, Christopher D. Graham, Barbara J. Klocke, Steven L. Carroll, Kevin A. Roth

**Affiliations:** Department of Pathology, University of Alabama at Birmingham, Birmingham, Alabama, United States of America; University of Manitoba, Canada

## Abstract

Malignant peripheral nerve sheath tumors (MPNSTs) are aggressive Schwann cell-derived sarcomas and are the leading cause of mortality in patients with neurofibromatosis type 1 (NF1). Current treatment modalities have been largely ineffective, resulting in a high rate of MPNST recurrence and poor five-year patient survival. This necessitates the exploration of alternative chemotherapeutic options for MPNST patients. This study sought to assess the cytotoxic effect of the BH3-mimetic AT101 [(-)-gossypol] on MPNST cells *in vitro* and to identify key regulators of AT101-induced MPNST cell death. We found that AT101 caused caspase-independent, non-apoptotic MPNST cell death, which was accompanied by autophagy and was mediated through HIF-1α induced expression of the atypical BH3-only protein BNIP3. These effects were mediated by intracellular iron chelation, a previously unreported mechanism of AT101 cytotoxicity.

## Introduction

Malignant peripheral nerve sheath tumors (MPNSTs) are highly aggressive Schwann cell-derived sarcomas that frequently arise from benign plexiform neurofibromas [1]. About half of all MPNSTs arise sporadically, with the remainder occurring in patients with neurofibromatosis type 1 (NF1) [2]. NF1 is the most commonly inherited autosomal dominant disorder affecting the human nervous system, occurring in 1 in 3500 newborn infants, and 8–13% of NF1 patients will develop MPNSTs in their lifetime [3]. The current standard of treatment for MPNSTs is surgery, however, complete surgical resection is often not possible and MPNSTs aggressively invade adjacent tissues and metastasize [4]. Radiation therapy inhibits local MPNST recurrence but does not increase patient survival. Chemotherapy is similarly ineffective. Consequently, MPNSTs are the leading cause of death in NF1 patients [5]. Given these limited treatment options and the aggressive behavior of MPNSTs, new therapeutic approaches are needed.

The ability to resist cell death by evading apoptosis is a hallmark of neoplastic cells [6]. This cancer cell characteristic reflects, in part, a dysregulation of the balance between pro-apoptotic and anti-apoptotic BCL-2 proteins [7] that regulate the intrinsic mitochondrial apoptotic pathway. The anti-apoptotic members of the BCL-2 family (BCL-2, BCL-xL, BCL-w, MCL-1 and A-1) inhibit apoptosis by binding to the multi-domain, pro-apoptotic members (BAX and BAK), which are embedded in the mitochondrial outer membrane. Many cancers overexpress anti-apoptotic members of the BCL-2 family and are resistant to death stimuli [8]. MPNSTs express high levels of BCL-xL, as compared to plexiform neurofibromas, which is thought to contribute to their chemoresistance [9]. Moreover, inhibition of BCL-xL sensitizes NF1-derived MPNST cells to chemotherapy [10].

AT101 [(-)-gossypol acetic acid] is a modified levo-enantiomer of gossypol, a naturally occurring polyphenolic aldehyde present in cottonseeds [11]. Although the use of gossypol as an antineoplastic agent has been explored since the mid 1960's [12], [13], it received renewed interest as a viable anti-cancer drug in the early 2000's when it was found to be a BH3-mimetic [14]. BH3-mimetics act like BCL-2 homology domain 3-only (BH3-only) proteins and interact with the BH3 binding groove of anti-apoptotic BCL-2 proteins; thereby preventing their interaction with the pro-apoptotic BCL-2 family proteins, Bax and Bak. It has been previously shown that gossypol inhibits the anti-apoptotic function of BCL-2, BCL-xL and MCL-1 [15]. Interestingly, gossypol was also previously tested as a male anti-fertility agent in clinical trials [16], [17]. The anti-fertility action of gossypol was thought to be due to inhibition of the cellular energy metabolism of spermatogonia [18] rather than its action as a BH3-mimetic. Several other modes of cytotoxic action have also been attributed to gossypol including inhibition of DNA synthesis and cell cycle arrest [19], altered intracellular calcium regulation [20], inhibition of protein kinase C [21], interaction with steroid receptor co-activators [22] and modulation of several components of mitochondrial apoptotic signaling [23]. There is also recent evidence that gossypol can induce autophagy, which in some cases leads to autophagic cell death [24]. Given these multiple actions, the primary mechanism by which gossypol induces cell death in any particular tumor type is likely to vary, depending on the levels of anti-apoptotic BCL-2 proteins and other tumor cell-specific factors.

The goal of this study was to determine whether AT101 exerts a cytotoxic effect on MPNST cells *in vitro* and to investigate its potential molecular mechanisms of action. We found that AT101 causes caspase-independent, non-apoptotic cell death in MPNST cells accompanied by autophagy which was not cytoprotective. We show that AT101 induces expression of BCL-2/E1B-19K-interacting protein 3 (BNIP3), an atypical BH3-only protein, which promotes AT101-induced cell death. We also demonstrate that BNIP3 expression is promoted by intranuclear accumulation of Hypoxia Inducible Factor-1α (HIF-1α), which can be abrogated by iron supplementation. Our findings further argue that one mechanism of AT101 cytotoxicity is chelation of intracellular iron.

## Materials and Methods

### Antibodies and Other Reagents

Primary antibodies were obtained from the following sources: HIF-1α, BECLIN1, BIM, BCL-xL, β-tubulin, Ferritin HC and transferrin receptor-1 (TfR1/CD71; Santa Cruz Biotechnology Inc., Santa Cruz, CA); BNIP3 (Sigma, St. Louis, MO); Lamin A/C (BD Transduction Laboratories, Franklin Lakes, NJ); GAPDH, BCL-2 and PUMA (Cell Signaling, Danvers, MA); LC3 (Abgent, San Diego, CA). Horseradish peroxidase (HRP)-conjugated goat anti-rabbit and horse anti-mouse antibodies were obtained from Biorad (Hercules, CA) and Cell Signaling (Danvers, MA), respectively.

AT101 was provided by Ascenta Therapeutics (Malvern, PA). BOC-aspartyl (Ome)-fluoromethyl ketone (BAF) and ferric citrate were purchased from MP Biomedicals (Aurora, OH), Bafilomycin A1 (BafA1) was from A.G. Scientific (San Diego, CA) and 3-methyladenine (3MA), Staurosporine (STS), Deferoxamine Mesylate (DFO), Dimethyl Sulfoxide (DMSO) from Sigma (St. Louis, MO). ABT-737 was purchased from Selleck Chemicals (Houston, TX).

### Cell Culture

We have previously described the source of T265-2c cells, ST88-14 cells and 90-8 cells, the human NF1-associated MPNST lines used in this study [25]. The identity of these cell lines was routinely verified according to the specifications outlined in the ATCC Technical Bulletin 8. Briefly, morphology and doubling times of cells were routinely assessed and the identity of cells was verified by short tandem repeat analyses. Cells were also regularly tested for Mycoplasma infection. All cell lines were cultured in DMEM10 [DMEM (Sigma, St. Louis, MO)] containing 1% penicillin/streptomycin (Invitrogen, Carlsbad, CA), 1% L-glutamine (Sigma, St. Louis, MO), and 10% fetal bovine serum (FBS; Hyclone, Logan, UT) and incubated at 37°C in a humidified 5% CO_2_, 95% air atmosphere. Cells were plated on uncoated 48 well plates at a density of 20,000/well and in 100 mm dishes at a density of 10^6^ cells/dish. Cultures were used in experiments 24 hours post-plating. Drug treatments were performed in media supplemented with 5% FBS. Concentrated stocks of AT101 (5 mM) were prepared in DMSO.

### Cell Viability, Cytotoxicity, Apoptosis and In Vitro Caspase Cleavage Assays

The calcein-AM (Life Technologies, Carlsbad, CA) conversion assay was used to measure cell viability. Caspase activation was assessed with an *in vitro* caspase-3 cleavage assay utilizing the chemical substrate DEVD-7-amino-4-methylcoumarin (AMC) (Enzo Life Sciences, Framingdale, NY). Both of these assays were performed as described previously [26]. Cytotoxicity and apoptosis were assessed using the *LIVE/DEAD* Viability/Cytotoxicity kit for mammalian cells and the Dead Cell Apoptosis kit with Annexin V Alexa Fluor 488 and Propidium Iodide from Molecular Probes Inc (Eugene, OR). Manufacturers' instructions were followed and ethidium homodimer-1, Alexa Fluor 488 and Propidium Iodide fluorescence were measured using a BD FACSCalibur flow cytometer.

### Western Blotting

Whole cell lysates were prepared by removing the media, washing the cells with PBS, scraping the cells free with a cell lifter and then pelleting them by centrifugation at 3000 rpm for 10 minutes. Cell pellets were resuspended in lysis buffer containing 20 mM Tris-HCl (pH 7.4), 150 mM NaCl, 2 mM EDTA, 1% Triton X-100, 10% glycerol, protease inhibitor cocktail (Sigma, St. Louis, MO), and phosphatase inhibitor cocktails 1 and 3 (Sigma, St. Louis, MO). Lysates were clarified and stored at −80°C. 30 µg of protein was immunoblotted per our previously described protocol [27]. All primary antibodies were diluted to a final concentration of 1∶1000 except GAPDH and LAMIN (1∶5000). Immunoreactive species were detected by enhanced chemiluminescence (Pierce ECL; Thermo Scientific, Waltham, MA). Nuclear and cytoplasmic fractions were extracted using the NE-PER extraction kit (Thermo Scientific, Waltham, MA) following the manufacturers' instructions.

### Immunocytochemistry

Primary antibodies were used at the following working concentrations: LC3 (Abgent; 1∶1000), BNIP3 (Sigma; 1∶100). HRP–conjugated anti-rabbit Super Picture (Invitrogen; used for LC3), and anti-mouse ImmPRESS (Vector Laboratories; used for BNIP3) were both used at 1∶100 dilutions. Immunoreactivity was detected using a tyramide signal amplification system using Cy3 (Perkin-Elmer Life Science Products). Bisbenzimide (1 µg/mL; Hoechst 33258; Sigma) was used for nuclear counterstaining. Samples were examined using a Zeiss Axioskop fluorescent microscope equipped with an AxioCam digital camera. Images were captured and analyzed using Axio Vision Rel. 4.8 software (Carl Zeiss MicroImaging).

### Reverse transcription and real-time quantitative PCR

RNA was isolated using the RNeasy Plus mini kit (Qiagen). cDNA was subsequently synthesized using a High Capacity cDNA Reverse Transcription kit (Applied Biosystems). Real-time quantitative PCR assays were performed using the Maxima SYBR Green/ROX qPCR master mix (Thermo Scientific, Waltham, MA) and the probes were obtained from Life Technologies (Carlsbad, CA). Amplifications were run in a Stepone Plus Real-Time PCR System (Applied Biosystems). The following sense/antisense primers and probes were selected from the Harvard PrimerBank and used for detecting human *BNIP3* (5′-TGAGTCTGGACGGAGTAGCTC-3′ and 5′-CCCTGTTGGTATCTTGTGGTGT-3′), *HIF-1α* (5′-GAACGTCGAAAAGAAAAGTCTCG-3′ and 5′-CCTTATCAAGATGCGAACTCACA-3′) and *ACTIN* (5′-GTCTGCCTTGGTAGTGGATAATG-3′ and 5′-TCGAGGACGCCCTATCATGG-3′). Analysis was done using the ΔΔC_T_ method (Livak and Schmittgen, 2001) and values were adjusted using *ACTIN* RNA levels as reference.

### RNAi

*BNIP3* and *HIF-1α* siRNA (siGENOME SMARTPool) was purchased from Thermo Scientific (Waltham, MA) and reconstituted according to the manufacturers' instructions. Cells were plated in DMEM10 and transfected 24 hours post-plating using X-tremeGene siRNA transfection reagent (Roche, Indianapolis, IN) at a ratio of 5∶2 [transfection reagent (µl):oligos (µg)]. The next day, fresh media was added to cells. Transfected cells were replated 48 hours post-transfection and treated 72 hours post-transfection.

### Iron Binding Assay

The iron binding properties of AT101, ABT737 and DFO were assessed in a cell free system using a modified protocol with the Iron/TIBC reagent set from Teco Diagnostics (Anaheim, CA). Briefly, the protocol for measuring unsaturated iron-binding capacity (UIBC) was modified to assess the iron binding capacity of the respective drugs in solution. Equal volumes of the UIBC buffer (Tris buffer 0.5 M, pH 8.0 with surfactant and sodium azide) were added to all wells followed by equal volumes of the iron standard supplied with the kit, except the blank. Various concentrations of AT101, ABT737 and DFO were added to their respective wells and volumes balanced with iron-free water. After the baseline absorbance was read at 560 nm, equal volumes of iron color reagent (Ferrozine 16.6 mM in hydroxylamine hydrochloride) were added to all wells and the plate incubated at 37°C for 10 minutes. Following incubation, absorbance was read again at 560 nm. The amount of iron bound was calculated using the manufacturers' instructions.

### Statistics

All data points represent mean ± S.D. All experiments were repeated at least 3 times unless stated otherwise. Representative data is shown. Statistical significance was determined by ANOVA followed by Bonferroni's posthoc test for multiple comparisons and student's t-test for two groups using Graphpad Prism 6 software. A *p*-value <0.05 was considered significant.

## Results

### AT101 induces caspase-independent cell death in MPNST cells

**Figure 1:**
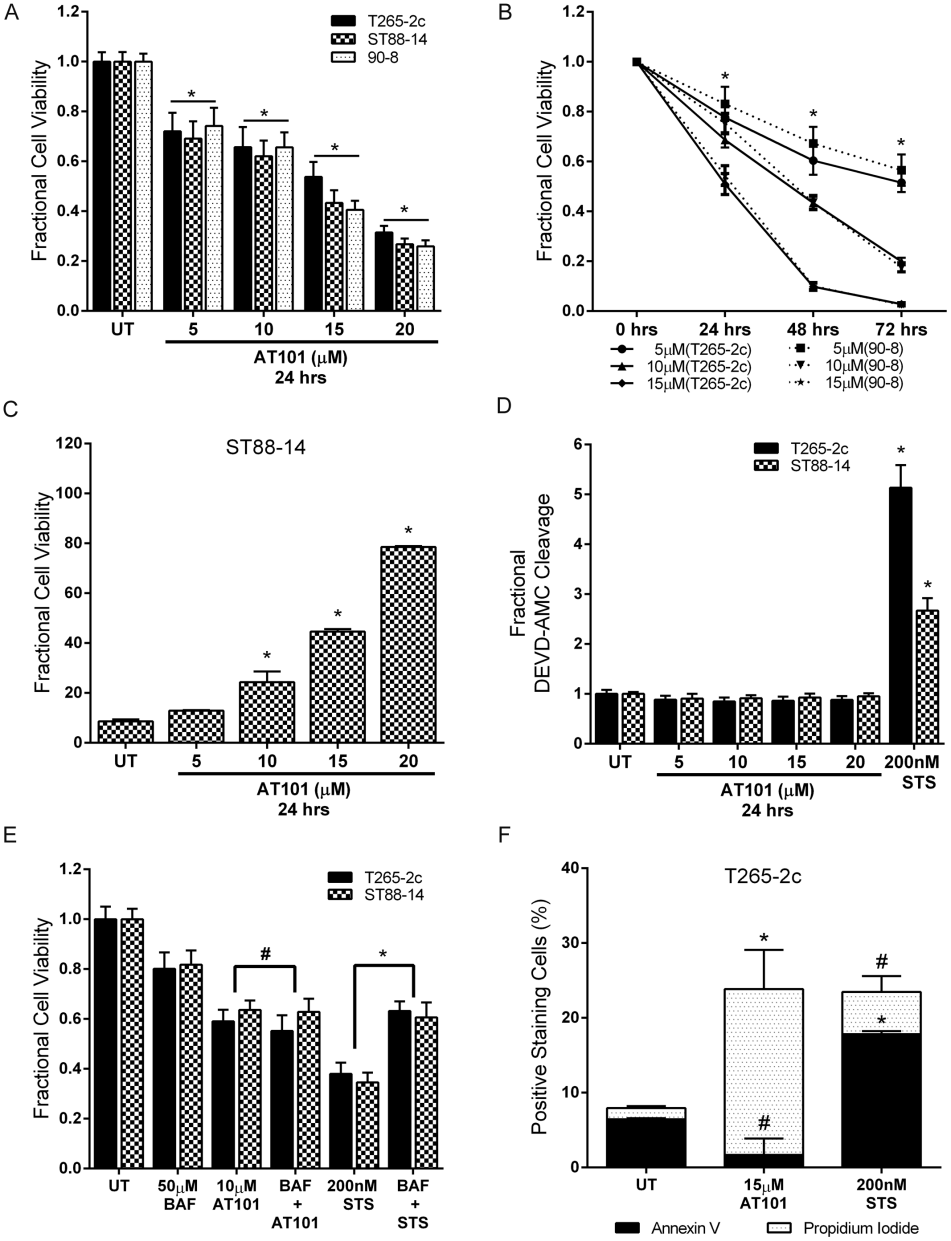
AT101 causes caspase-independent, non-apoptotic cell death in MPNST cells. AT101 treated cells demonstrate a concentration- and time-dependent decrease in viability (A, B). AT101 treatment causes concentration-dependent cytotoxicity in ST88-14 cells (C). The loss of cell viability is not accompanied by increased caspase-3 like enzymatic activity (D) and broad caspase inhibition with BAF did not protect from AT101-induced cell death (E). AT101 (15 µM for 24 hours) treatment does not increase annexin V staining in T265-2c cells (F). Staurosporine (STS) was used as a positive control for induction of caspase-3 like activity, annexin V staining and caspase-dependent apoptosis. * *p*-value <0.05. #  =  Not significant, *p*-value >0.05.

The effects of AT101 on human MPNST cells were assessed using NF-1 patient-derived MPNST cell lines - (T265-2c, ST88-14 and 90-8 cells). To determine whether AT101 inhibits MPNST viability, cells were treated for 24–72 hours with 5–20 µM AT101 and cell viability assessed using calcein-AM cleavage assays. We found that AT101 decreased MPNST cell viability in a concentration- and time-dependent manner (Fig.1A–B). We confirmed that the vehicle (DMSO), at the concentrations used (1–4 µl/ml media), had no effect on cell viability (Fig. S1A, B). Since (-)-gossypol has been reported to induce growth inhibition in cancer cells [28] and the calcein-AM cleavage assay measures only viable cells, we assessed AT101 induced cell death by measuring ethidium homodimer-1 staining in treated cells by flow cytometry. AT101 treatment resulted in a concentration-dependent increase in the percentage of ethidium homodimer-1 positive dead ST88-14 cells (Fig.1C; similar results were obtained in two other cell lines; data not shown).

Cancer cell death induced by (-)-gossypol can result from either caspase-dependent or caspase-independent mechanisms [24], [29]. To determine whether AT101 triggered effector caspase activation in MPNST cells, the cleavage of DEVD-AMC, a pharmacological substrate for active effector caspases (caspases-3, 6 and 7), was measured in AT101 treated cells after lysis. We found that AT101 treatment had no effect on caspase-3 like enzymatic activity in T265-2c and ST88-14 cells (Fig.1D). Consistent with these results, pretreatment with the broad spectrum caspase inhibitor, BAF, did not attenuate AT101-induced cytotoxicity in T265-2c and ST88-14 cells in contrast with its ability to inhibit staurosporine-induced death in these cells (Fig.1E). To further ascertain if AT101-induced MPNST cell death was apoptotic, we measured annexin V and propidium iodide staining in treated T265-2c cells by flow cytometry. AT101 treatment did not increase annexin V positive cells while increasing propidium iodide positive cells (Fig.1F). Based on these findings, we conclude that AT101 causes MPNST cell death via a mechanism other than caspase-dependent apoptosis.

### Autophagy triggered by AT101 during MPNST cell death is not cytoprotective

**Figure 2:**
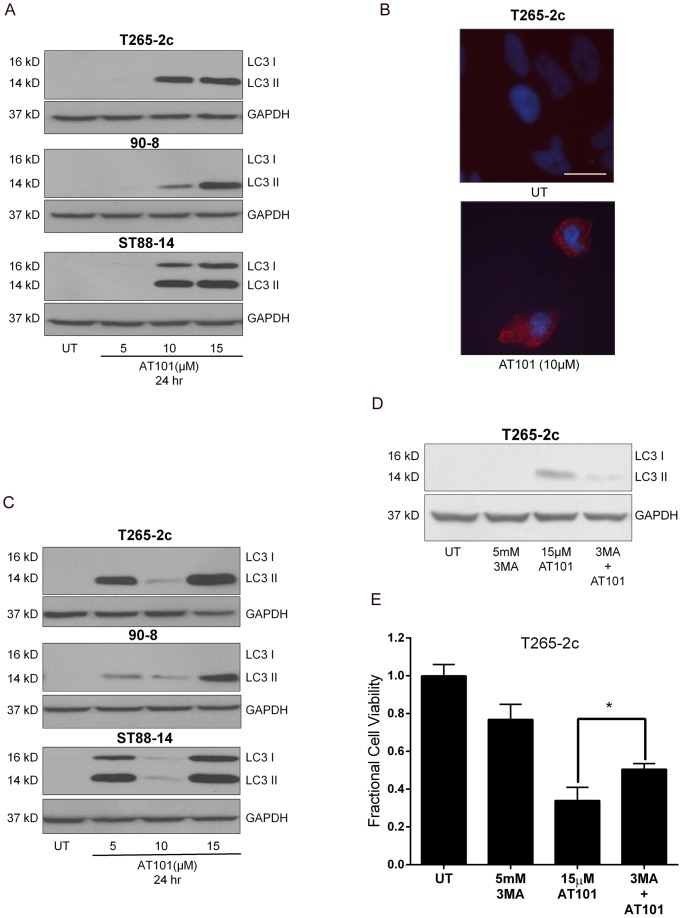
AT101-induced autophagy in MPNST cells is not cytoprotective. Treatment with AT101 leads to a concentration-dependent increase in steady state levels of LC3-II in MPNST cells (A) and a punctate LC3-like immunostaining pattern indicative of AV accumulation (B). AT101 causes an increase in autophagic flux in MPNST cells as evidenced by increased LC3 II levels in cells treated with both AT101 (10 µM for 24 hours) and BafA1 (100 nM, added 4 hours before collection of lysates) compared to either drug alone (C). T265-2c cells treated with 3-Methyladenine (3 MA–5 mM one hour prior to treatment with AT101) showed decreased level of LC3-II accumulation (D) and a modest but statistically attenuation of AT101 (15 µM for 24 hours) -induced cell death (E). Scale bar = 20 microns. * *p*-value <0.05.

(-)-Gossypol has been previously shown to stimulate autophagy in other cancer types [30], [31]. However, both cytoprotective and cytotoxic roles have been ascribed to (-)-gossypol induced autophagy in cancer cells [24], [31]. Consequently, we first asked whether AT101 also stimulates autophagy in MPNST cells. Whole cell lysates of AT101 treated cells were immunoblotted and probed for changes in levels of LC3 II, a well accepted surrogate marker of autophagic vacuoles (AVs) [32]. Relative to untreated controls, AT101 treated cells demonstrated a dramatic increase in steady state levels of LC3 II (Fig.2A). We confirmed this observation by performing immunocytochemistry, which demonstrated that AT101 treatment altered the intracellular distribution of LC3-like immunoreactivity from a relatively weak, diffuse staining pattern to a strong punctate pattern consistent with enhanced AV content in T265-2c cells (Fig.2B).

Although an increase in LC3 II levels indicates accumulation of AVs, this can reflect increased autophagy induction and/or decreased AV degradation. We therefore assessed autophagic flux in MPNST cells treated with AT101 in the presence or absence of Bafilomycin A1 (BafA1), a vacuolar ATPase inhibitor that blocks AV degradation [33]. We observed increased LC3 II levels in cells treated with both AT101 and BafA1 compared to either drug alone, indicating AT101 stimulated AV formation (Fig.2C).

To assess the functional role of AT101-induced autophagy in MPNST cells, we inhibited AV formation by treating cells with 3-Methyladenine (3MA) (Fig.2D) prior to exposure to AT101. 3MA is an inhibitor of class III phosphatidylinositol 3-kinase, which is required for the initiation of autophagy [34], [35]. We found that pharmacological inhibition of autophagy with 3MA resulted in a modest but statistically significant attenuation in AT101-induced T265-2c cell death (Fig.2E). These findings indicate that AT101-induced autophagy in MPNST cells is not cytoprotective and could potentially play a cytotoxic role.

### BNIP3 mediates AT101-induced cell death in MPNST cells

BNIP3 is an atypical BH3-only protein involved in cell death, autophagy and mitochondrial clearance [36]. BNIP3 can mediate a non-apoptotic form of cell death, which is caspase-independent and associated with induction of autophagy, in response to some stimuli [37]–[39]. These observations led us to hypothesize that BNIP3 mediates AT101-stimulated MPNST cell death.

**Figure 3:**
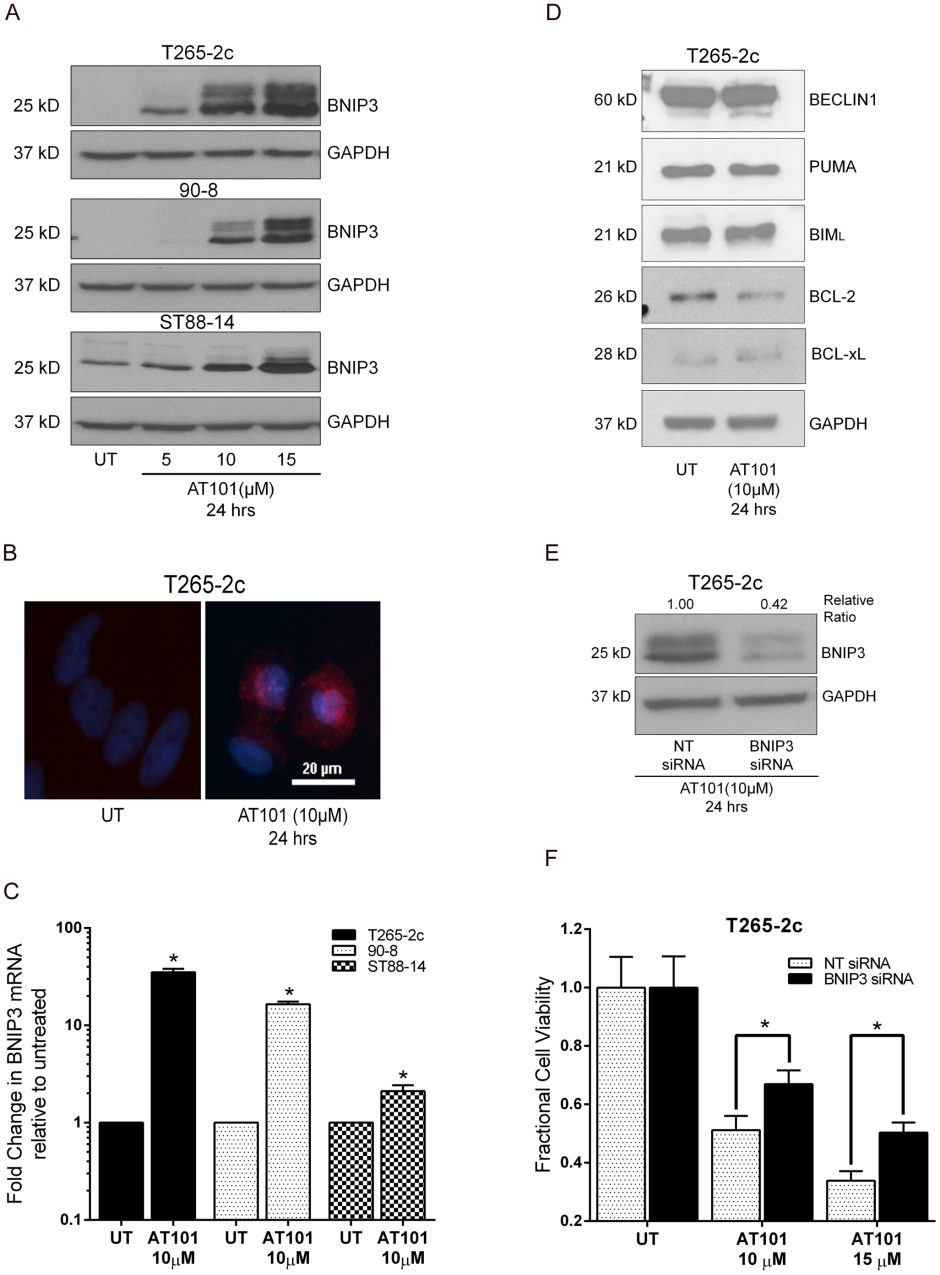
BNIP3 regulates AT101-induced cytotoxicity in MPNST cells. AT101 treated MPNST cells show a concentration-dependent increase in BNIP3 protein on western blot (A) and immunocytochemistry (B). AT101 treatment causes a significant increase in BNIP3 mRNA in MPNST cells compared to untreated cells (C). AT101 (10 µM for 24 hours) does not significantly alter the expression of BCL-2, BCL-xL, BIM, PUMA, and BECLIN1 protein levels in T265-2c cells (D). T265-2c cells transfected with siRNA against *BNIP3* showed a significant decrease in BNIP3 protein levels (relative ratio to GAPDH) after treatment with AT101 (E) and were significantly protected from AT101-induced cell death (F), relative to cells transfected with non-target siRNA. Scale bar  = 20 microns. * *p*-value <0.05.

We first asked whether BNIP3 protein levels are increased in MPNST cells treated for 24 hours with 5–20 µM AT101. Immunoblot analyses of whole cell lysates demonstrated that AT101 treatment increased levels of BNIP3 protein in a concentration-dependent manner (Fig.3A). Immunocytochemistry similarly showed an increase in BNIP3-like immunoreactivity in AT101-treated MPNST cells (Fig.3B). To determine if the increase in BNIP3 protein is associated with a corresponding increase in mRNA, we used quantitative real time PCR to assess the steady state levels of BNIP3 mRNA in MPNST cells after treatment with AT101. We found that AT101 treatment resulted in a significant increase in BNIP3 mRNA levels when compared to untreated MPNST cells (Fig.3C).

As (-)-gossypol has been reported to affect the protein levels of some BCL-2 family members, we examined the levels of BCL-2, BCL-xL, BIM, PUMA and BECLIN1 in T265-2c cells following AT101 treatment. We found that the levels of these proteins were largely unaffected by AT101 treatment (Fig.3D). Therefore, we focused on the functional role of AT101-induced BNIP3 expression in MPNST cells. To determine whether BNIP3 promotes AT101-induced MPNST cell death, we transiently knocked down BNIP3 expression in T265-2c cells by transfecting them with BNIP3 siRNA or a control non-targeting siRNA. We found that the BNIP3, but not the control, siRNA effectively attenuated BNIP3 expression (Fig.3D). We then treated T265-2c cells with 10 or 15 µM AT101 and compared cell viability in the BNIP3 siRNA transfected cells to that in cells transfected with control oligonucleotide. We found that AT101-induced cell death was significantly attenuated in cells with BNIP3 knockdown in comparison to cells transfected with non-targeting siRNA (Fig.3E). We conclude that BNIP3 is an important mediator of AT101-induced MPNST cytotoxicity.

### AT101 causes accumulation of HIF-1α protein, which regulates BNIP3 expression

**Figure 4:**
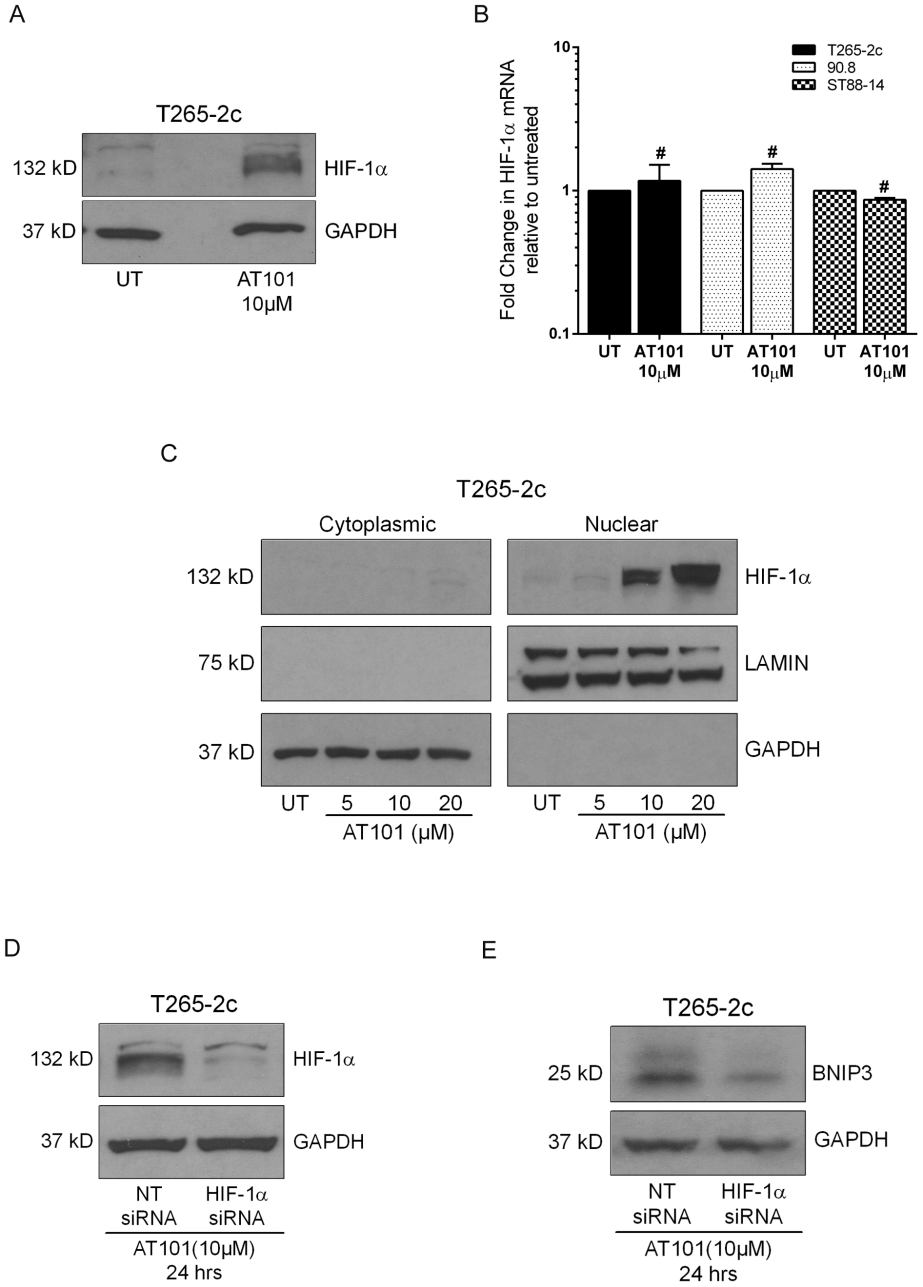
AT101 stimulates BNIP3 expression through HIF-1α accumulation. T265-2c cells treated with AT101 (10 µM for 24 hours) demonstrate increased levels of HIF-1α protein (A), but AT101 treated MPNST cells do not show a significant change in HIF-1α mRNA levels (B) relative to untreated cells. AT101 causes an increase in HIF-1α protein levels in the nuclear fraction of T265-2c cells (C). T265-2c cells transfected with HIF-1α siRNA show decreased levels of HIF-1α protein (D) and BNIP3 protein expression (E) upon treatment with AT101, relative to cells transfected with non-target siRNA. #  =  Not significant.

BNIP3 is a direct transcriptional target of HIF-1α, which has been shown to regulate BNIP3 expression in response to hypoxia [40] and in glioma cells treated with ceramide [41]. HIF-1α can accumulate in conditions of hypoxia and following other non-hypoxic stimuli [42]. Accumulation of HIF-1α protein levels with AT101 or gossypol treatment has not been previously reported. Therefore, we examined the protein levels of HIF-1α in AT101-treated MPNST cells. We found that AT101 treatment increased the level of HIF-1α protein in T265-2c cells (Fig.4A) without an accompanying increase in HIF-1α mRNA (Fig.4B). We also found that following AT101 treatment, HIF-1α protein accumulates in the nucleus (Fig.4C) where it has been shown to function as a transcription factor. To determine whether HIF-1α transcriptionally regulates BNIP3 expression, we transiently knocked down HIF-1α expression in T265-2c cells (Fig.4D) and examined the effect on BNIP3 expression following AT101 treatment. We found that HIF-1α siRNA, but not control siRNA, inhibited BNIP3 expression in AT101-treated cells (Fig.4E). We conclude that AT101 causes an accumulation of HIF-1α protein in MPNST cells which in turn promotes the expression of BNIP3.

### AT101 causes accumulation of HIF-1α through intracellular iron chelation

**Figure 5:**
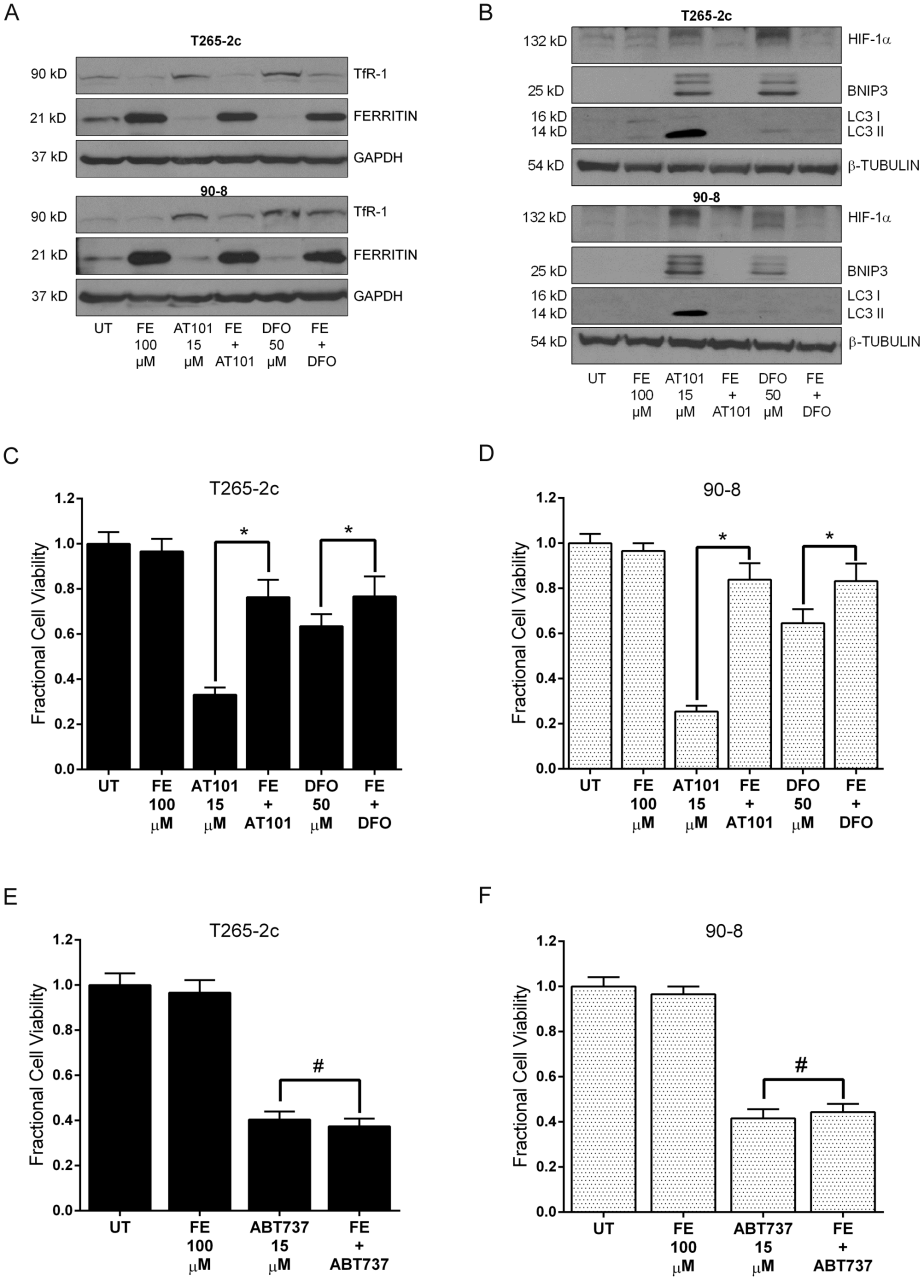
Iron supplementation protects MPNST cells from AT101-induced cytotoxicity. AT101 (15 µM for 24 hours) treatment increases transferrin receptor 1 (TfR1) protein levels and decreases ferritin protein levels in MPNST cells (A), which are reversed by addition of ferric citrate to the media. Supplementation of the culture media with ferric citrate (100 µM) also abrogates AT101-induced HIF-1α, BNIP3 and LC3II protein expression (B) and cytotoxicity in T265-2c and 90-8 cells (C, D). MPNST cells are not rescued from ABT737 (15 µM) induced cytotoxicity by supplementation of culture media with ferric citrate (100 µM) (E, F). DFO (50 µM for 24 hours) was used as a positive control for iron chelation. * *p*-value <0.05. #  =  Not significant.

In addition to hypoxia, certain non-hypoxic stimuli such as estrogen, insulin and epidermal growth factor [43]–[45], can induce HIF-1α expression in normoxic conditions. Interestingly, polyphenols like quercetin and galangin can also induce HIF-1α protein accumulation without increasing HIF1-α mRNA levels [46], much as we observed with AT101. This action is thought to be mediated via intracellular iron chelation [46], [47]. Therefore, we next asked whether iron was involved in AT101-induced HIF-1α accumulation, BNIP3 expression and MPNST cell death. To address if AT101 interfered with intracellular iron levels, we assessed the protein levels of transferrin receptor 1 (TfR1) and ferritin (heavy chains) in MPNST cells treated with AT101. TfR1 is a carrier protein that facilitates iron transport into the cell [48]. TfR1 expression is post-transcriptionally regulated and inversely related to intracellular iron concentration. Additionally, ferritin heavy chains catalyze the first step in intracellular iron storage and its protein levels correspond to intracellular iron levels [48]. We found that AT101 treatment increased TfR1 protein levels and decreased ferritin heavy chain protein levels in T265-2c and 90-8 cells and these AT101 effects were blocked when the media was supplemented with ferric citrate (100 µM), as a source of iron ions (Fig.5A). To determine if iron chelation was the mechanism through which AT101 induced an accumulation of HIF-1α and subsequently BNIP3 and autophagy, we assessed the effect of iron supplementation on these events. Addition of ferric citrate to the cell culture media abrogated the accumulation of HIF-1α, BNIP3 and LC3II induced by AT101 (Fig.5B). To further evaluate the functional relevance of iron supplementation on AT101-induced MPNST cytotoxicity, we assessed cell viability after addition of ferric citrate. We found that iron supplementation of the cell culture media provided significant protection from AT101-induced cytotoxicity in T265-2c and 90-8 cells (Fig.5C, D). These effects of AT101 treatment in MPNST cells were similar to those of DFO, a well-known iron chelator. Addition of ferric citrate to the cell culture media failed to affect the cytotoxic action of ABT737, a structurally distinct BH3-mimetic on T265-2c and 90-8 cells (Fig.5E, F).

**Figure 6:**
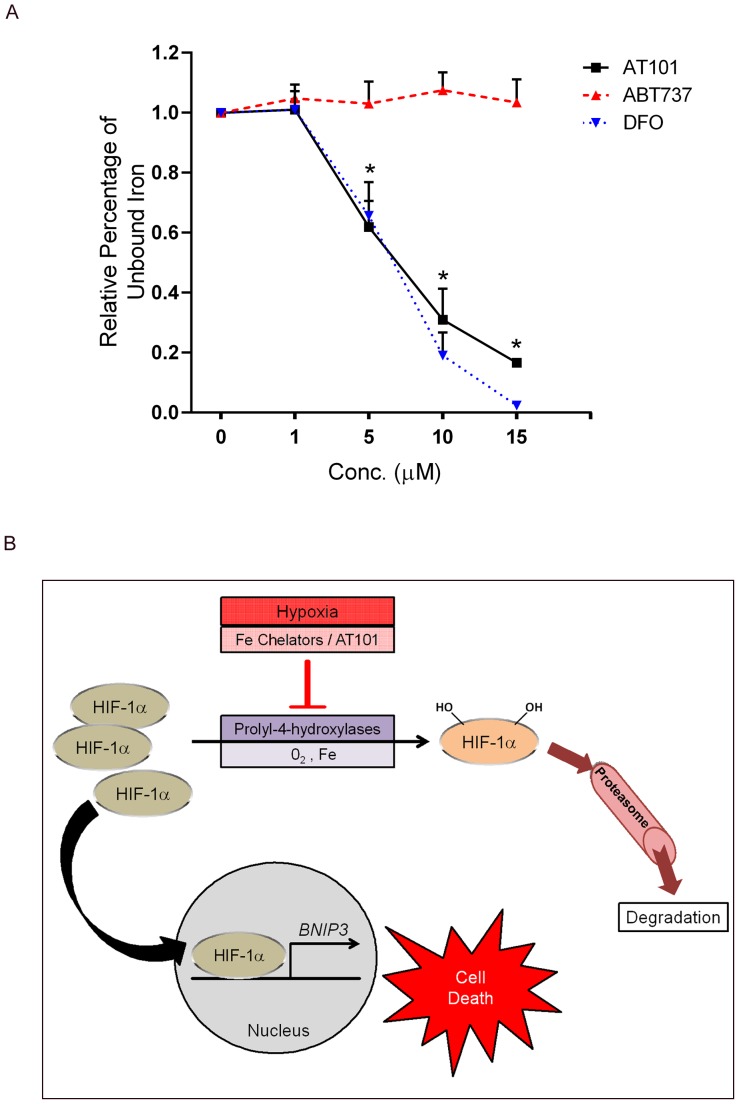
AT101 binds iron and acts as an iron chelator. AT101 and DFO (0.1–10 µM) can directly bind iron and reduce the absorbance due to free iron, whereas ABT737, at similar concentrations, does not bind iron and thus does not alter the absorbance * *p*-value <0.05. (A). Schematic representation of the proposed mechanism of action of AT101 in MPNSTs (B). In normoxia, Prolyl-hydroxylases (PHDs) play a critical role in HIF-1α degradation. AT101 chelates intracellular iron, which is required for the proper function of PHDs. Loss of function of the PHDs leads to an accumulation of HIF-1α, which translocates to the nucleus and transcriptionally upregulates BNIP3 expression leading to cell death.

To determine if AT101 produced these effects by directly binding iron, we measured unsaturated iron binding capacity in the presence or absence of AT101 in a cell free *in vitro* assay. We found that AT101 and DFO, but not ABT737, bound iron in a concentration dependent manner (Fig.6A). These observations indicate that AT101 induces HIF-1α accumulation and subsequent BNIP3 expression through chelation of intracellular iron, independent of its widely reported BH3-mimetic properties.

## Discussion

AT101 is a modified enantiomer of gossypol, a naturally occurring small molecule antagonist of the anti-apoptotic BCL-2 proteins [49], [50]. AT101 and its parent compound, gossypol, have been shown to have cytotoxic effects in a wide range of cancer cells and AT101 is currently in phase II clinical trials for several cancers [51]–[54]. In addition to being identified as a BH3-mimetic by computer-assisted molecular modeling, nuclear magnetic resonance imaging, and fluorescence-polarization assays, other studies have demonstrated that gossypol can inhibit DNA synthesis [19], cellular energy metabolism [55], [56], protein kinase C [21], telomerase [57] and the human mitotic kinesin, EG5 [58]. In addition to these actions, gossypol can modulate calcium homeostasis [20] and TGF-β1 signaling [59]. In the wake of the structural studies indicating that gossypol is a BH3-mimetic, it was found that gossypol can activate the intrinsic apoptotic pathway by increasing reactive oxygen species [60], activating p53 [61] and suppressing NF-κB activity [62]. In addition, gossypol can increase the expression of pro-apoptotic BH3-only proteins [23] and induce a conformational change in BCL-2 which renders it pro-apoptotic [63]. As the mechanism of gossypol's cytotoxic action is likely influenced by the genetic profile of the cancer cell and other tumor-specific factors, identifying the relevant mechanisms of AT101 cytotoxic action is important in assessing its potential use in MPNST treatment.

In this study, we focused on identifying the molecular regulators involved in AT101-induced MPNST cytotoxicity. We found that AT101 caused concentration-dependent MPNST cytotoxicity which was neither accompanied by increased annexin V staining or caspase-3 like enzymatic activity nor attenuated by broad caspase inhibition. This indicated that AT101-induced MPNST cell death was caspase-independent and could potentially involve a non-apoptotic death mechanism. Gossypol stimulates autophagy in multiple cancer types by disrupting the BCL-2/BECLIN1 complex [64] as well as through a BECLIN-independent mechanism [31]. Previously, (-)-gossypol has been shown to cause caspase-independent cytotoxicity in malignant gliomas via activation of autophagic cell death [24]. However, other studies have reported that gossypol stimulated autophagy is cytoprotective [31]. These conflicting observations suggest that the cytoprotective or cytotoxic effects of gossypol-induced autophagy may depend on the cancer type. We found that AT101 also stimulates autophagy in MPNST cells. Inhibition of AV formation with 3MA demonstrated that AT101-induced autophagy in MPNST cells was not cytoprotective. The slight attenuation of AT101-induced cytotoxicity after pharmacological inhibition of autophagic vacuole formation suggests that autophagy may make a modest contribution to AT101-induced MPNST cell death. Alternatively, AT101-induced autophagy could be producing a cytostatic response or could simply be a nonprotective increase in cellular degraditive functions [65].

Gossypol inhibits anti-apoptotic BCL-2 proteins by occupying their BH3 binding groove and can also modulate the expression of anti- and pro-apoptotic proteins [23]. Upon examining the effect of AT101 treatment on BCL-2 family protein expression in MPNST cells, we found that BNIP3 protein expression was markedly increased while expression of other BCL-2 proteins was not significantly altered. Interestingly, BNIP3 is a known mediator of caspase-independent, non-apoptotic cell death induced by various stimuli. Microarray analyses have previously shown that (-)-gossypol upregulates BNIP3 mRNA levels [66]. However, this previous study did not explore the mechanisms of gossypol-mediated induction of BNIP3 expression or the role BNIP3 plays in regulating gossypol-induced cytotoxicity.

In this study, we demonstrated that BNIP3 plays an important role in AT101-induced cytotoxicity. Although BNIP3 knockdown did not completely abolish AT101-induced death, this may be due to incomplete knockdown of BNIP3 protein or indicate that other cell death mediators also contribute to AT101 cytotoxicity. Our results corroborate and extend previous observations and further demonstrate that BNIP3 is a novel regulator of AT101-induced MPNST cytotoxicity.

Earlier studies in our laboratory and others have shown that BNIP3 can be transcriptionally regulated by HIF-1α [67], [68]. Although HIF-1α can be induced in normoxic conditions by various factors, gossypol or AT101 have not been previously shown to induce HIF-1α. We observed that AT101 caused an accumulation of HIF-1α protein that was not accompanied by an increase in HIF-1α mRNA levels. We also showed that HIF-1α translocates to the nucleus following AT101 exposure and demonstrated that knocking down HIF-1α decreases AT101-stimulated BNIP3 expression. Under normoxic conditions, the half-life of HIF-1α is only a few minutes as it is continually hydroxylated by prolyl-hydroxylases (PHDs) [42]. It is then recognized by von Hippel-Lindau protein, an E3 ubiqitin ligase, which targets HIF-1α for degradation through the proteasome pathway. As PHDs require oxygen and iron ions to maintain their enzymatic activity, hypoxia or chelation of intracellular iron can inhibit their function. Some polyphenols such as quercetin and galangin promote HIF-1α accumulation by chelating intracellular iron [46]. Gossypol is a naturally occurring polyphenol and shares structural similarities (in particular, the presence of catechol moieties) with quercetin and galangin. Interestingly, although studies of cotton seed fed farm animals have suggested that gossypol affects iron levels [69], gossypol has not been previously reported to bind iron. It is known that iron chelators like DFO act as hypoxia-mimetics and promote the accumulation of HIF-1α by rendering the PHDs dysfunctional. Polyphenols with catechol moieties have been reported to exert their actions through a similar mechanism [46]. We found that AT101 alters the expression of intracellular iron carrier and storage proteins in a way indicative of intracellular iron chelation. We demonstrated that AT101, like DFO, can directly bind iron in a cell free system and that a structurally unrelated BH3-mimetic, ABT737, failed to do so. Finally, these findings are consistent with our observation that iron supplementation inhibits AT101-induced HIF-1α accumulation, BNIP3 induction and autophagy. Consequently, our studies identify a previously unreported mechanism for AT101-induced cell death, namely an ability to chelate intracellular iron (Fig.6B).

Although we have not directly assessed the role of iron chelation in other reported actions of gossypol, it is well-recognized that iron is essential for the function of several proteins and enzymes involved in cell cycle progression, DNA synthesis and energy metabolism [19]. We hypothesize that intracellular iron chelation by AT101 could play a role in mediating these effects and contribute to the BNIP3-independent decrease in MPNST cell viability. Even though we focused our study specifically on MPNST cells, we also observed that AT101 induced significant BNIP3 expression in malignant glioma (U87) and breast cancer (MCF7) cell lines (data not shown) suggesting that a similar mechanism of AT101 action could contribute to cytotoxicity in other cancers. Future investigations into this possibility are needed to advance the therapeutic use of AT101 in MPNSTs and other cancers.

## Supporting Information

Figure S1 **DMSO does not affect MPNST cell viability.** MPNST cells treated with DMSO (1–4 µl/ml cell culture media) for 24 hours did not affect T265-2c and ST88-14 cell viability (A, B). #  =  Not significant, *p*-value >0.05.
(TIF)
