# Correction: BNIP3 Regulates AT101 [(-)-Gossypol] Induced Death in Malignant Peripheral Nerve Sheath Tumor Cells

**DOI:** 10.1371/journal.pone.0137153

**Published:** 2015-08-27

**Authors:** Niroop Kaza, Latika Kohli, Christopher D. Graham, Barbara J. Klocke, Steven L. Carroll, Kevin A. Roth

There is an error in Fig 1. In Fig 1C, the y-axis should read: “Percentage of ethidium homodimer-1 positive dead cells.” Please see the corrected Fig 1 here.

**Fig 1:**
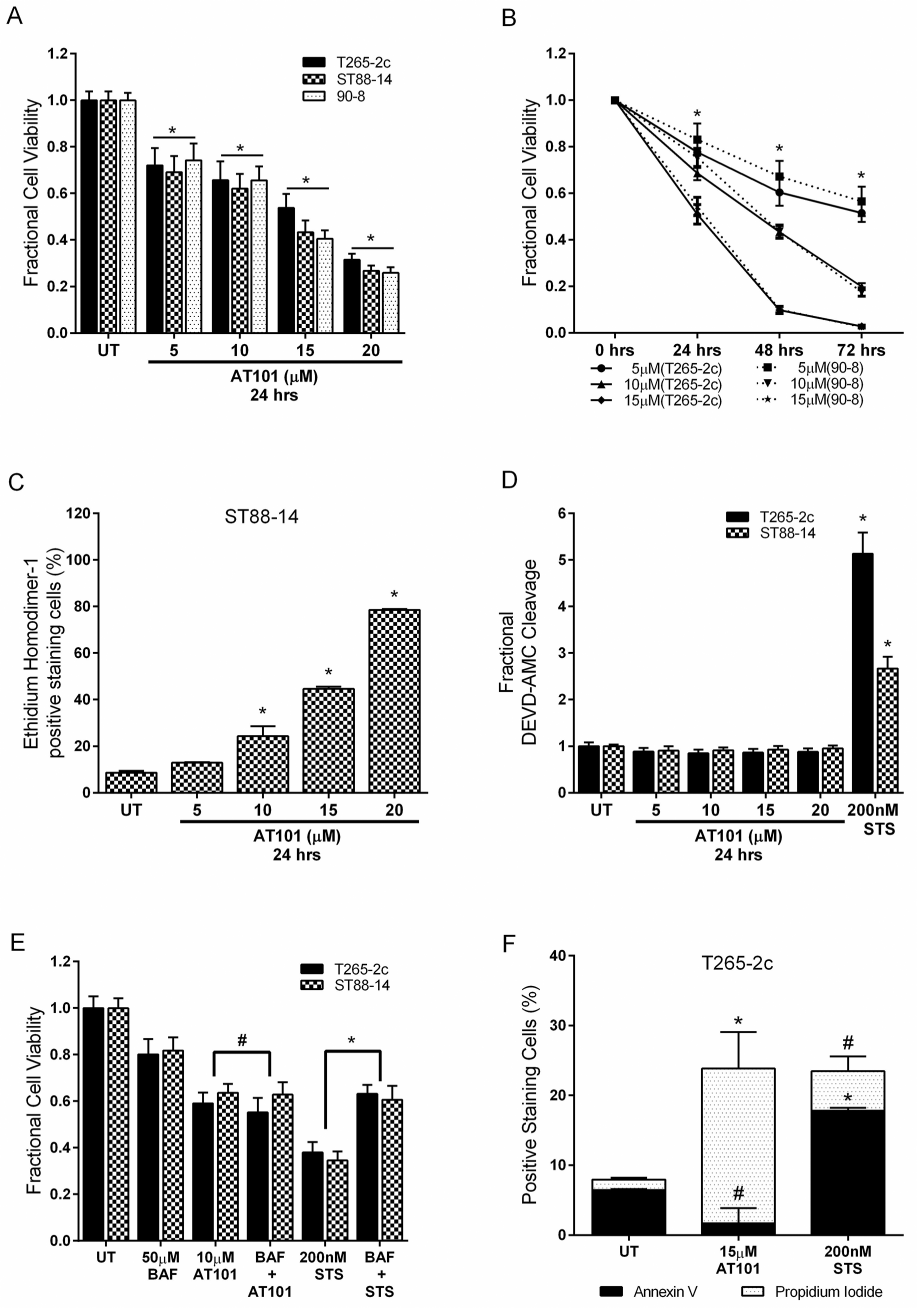
AT101 causes caspase-independent, non-apoptotic cell death in MPNST cells. AT101 treated cells demonstrate a concentration- and time-dependent decrease in viability (A, B). AT101 treatment causes concentration-dependent cytotoxicity in ST88–14 cells (C). The loss of cell viability is not accompanied by increased caspase-3 like enzymatic activity (D) and broad caspase inhibition with BAF did not protect from AT101-induced cell death (E). AT101 (15 μM for 24 hours) treatment does not increase annexin V staining in T265–2c cells (F). Staurosporine (STS) was used as a positive control for induction of caspase-3 like activity, annexin V staining and caspase-dependent apoptosis. * *p*-value <0.05. # = Not significant, *p*-value >0.05.

There are errors in Fig 2. In Fig 2C, the bands of the western blot are labelled incorrectly. Please see the corrected Fig 2 here.

**Fig 2:**
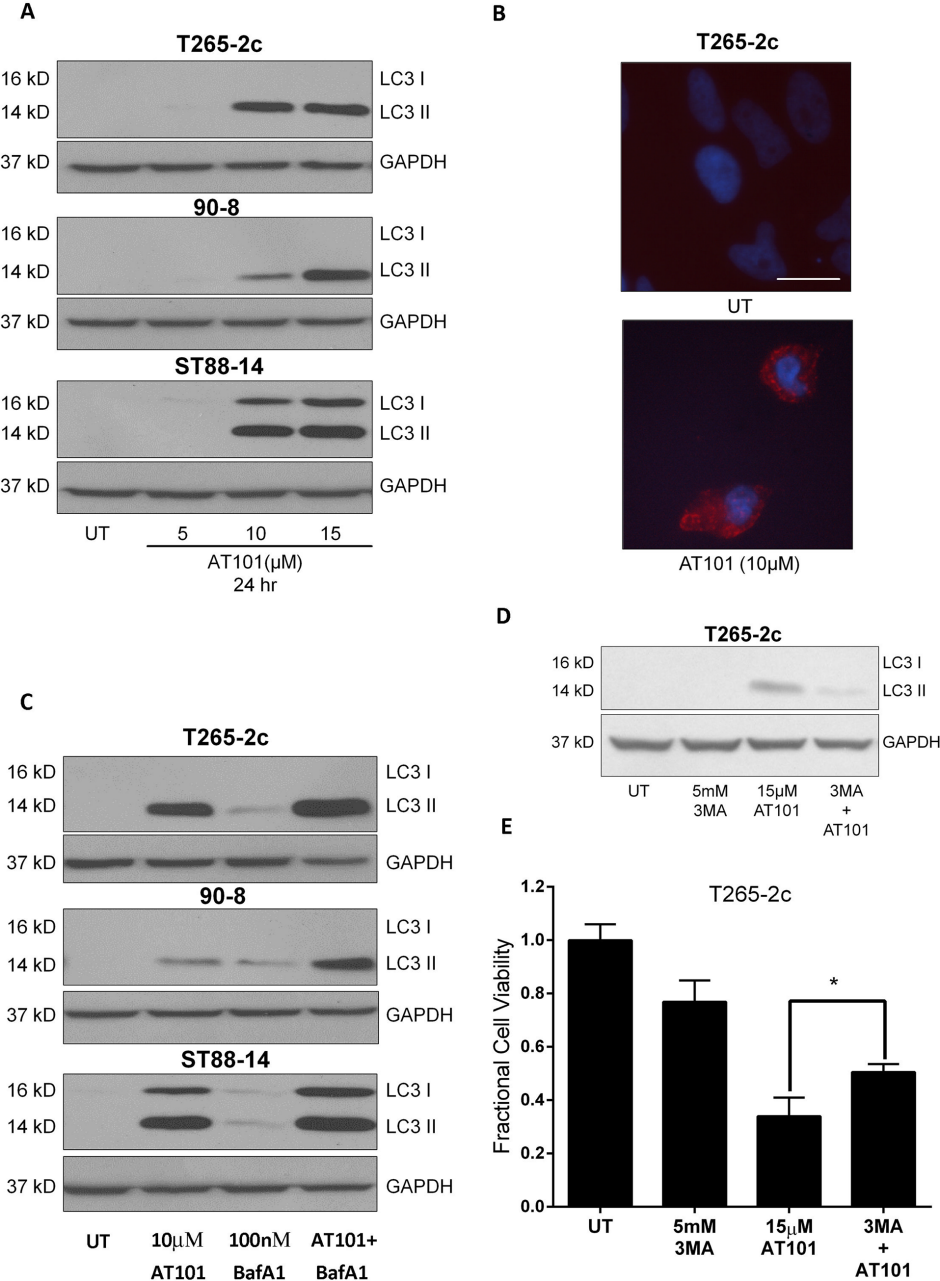
AT101-induced autophagy in MPNST cells is not cytoprotective. Treatment with AT101 leads to a concentration-dependent increase in steady state levels of LC3-II in MPNST cells (A) and a punctate LC3-like immunostaining pattern indicative of AV accumulation (B). AT101 causes an increase in autophagic flux in MPNST cells as evidenced by increased LC3 II levels in cells treated with both AT101 (10 μM for 24 hours) and BafA1 (100 nM, added 4 hours before collection of lysates) compared to either drug alone (C). T265–2c cells treated with 3-Methyladenine (3 MA–5 mM one hour prior to treatment with AT101) showed decreased level of LC3-II accumulation (D) and a modest but statistically attenuation of AT101 (15 μM for 24 hours)-induced cell death (E). Scale bar = 20 microns. * *p*-value <0.05.
