# Supplementary figures and images for: BNIP3 Regulates AT101 [(-)-Gossypol] Induced Death in Malignant Peripheral Nerve Sheath Tumor Cells
Source: PLoS One. 2014 May 13;9(5):e96733. doi: 10.1371/journal.pone.0096733 (PMC4019476; doi:10.1371/journal.pone.0096733)

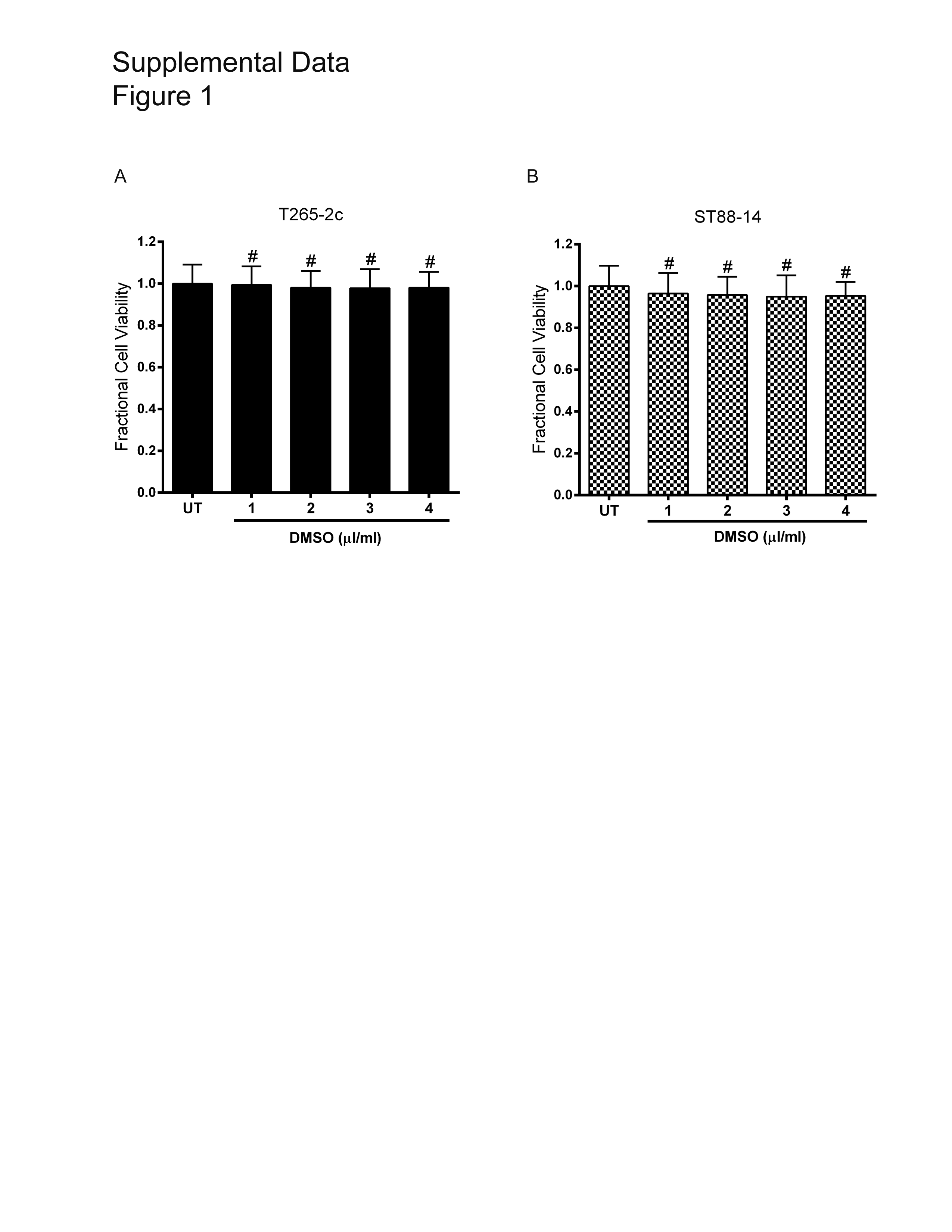

Supplement: Figure S1 — DMSO does not affect MPNST cell viability. MPNST cells treated with DMSO (1–4 µl/ml cell culture media) for 24 hours did not affect T265-2c and ST88-14 cell viability (A, B). # = Not significant, p-value >0.05. (TIF) [file pone.0096733.s001.tif]
